# Inhibiting Sperm Pyruvate Dehydrogenase Complex and Its E3 Subunit, Dihydrolipoamide Dehydrogenase Affects Fertilization in Syrian Hamsters

**DOI:** 10.1371/journal.pone.0097916

**Published:** 2014-05-22

**Authors:** Archana B. Siva, Subbarayalu Panneerdoss, Purnima Sailasree, Durgesh K. Singh, Duvurri B. Kameshwari, Sisinthy Shivaji

**Affiliations:** Centre for Cellular and Molecular Biology (Council of Scientific and Industrial Research), Hyderabad, India; University of Hyderabad, India

## Abstract

**Background/Aims:**

The importance of sperm capacitation for mammalian fertilization has been confirmed in the present study via sperm metabolism. Involvement of the metabolic enzymes pyruvate dehydrogenase complex (PDHc) and its E3 subunit, dihydrolipoamide dehydrogenase (DLD) in hamster *in vitro* fertilization (IVF) via *in vitro* sperm capacitation is being proposed through regulation of sperm intracellular lactate, pH and calcium.

**Methodology and Principal Findings:**

Capacitated hamster spermatozoa were allowed to fertilize hamster oocytes *in vitro* which were then assessed for fertilization, microscopically. PDHc/DLD was inhibited by the use of the specific DLD-inhibitor, MICA (5-methoxyindole-2-carboxylic acid). Oocytes fertilized with MICA-treated (MT) [and thus PDHc/DLD-inhibited] spermatozoa showed defective fertilization where 2^nd^ polar body release and pronuclei formation were not observed. Defective fertilization was attributable to capacitation failure owing to high lactate and low intracellular pH and calcium in MT-spermatozoa during capacitation. Moreover, this defect could be overcome by alkalinizing spermatozoa, before fertilization. Increasing intracellular calcium in spermatozoa pre-IVF and in defectively-fertilized oocytes, post-fertilization rescued the arrest seen, suggesting the role of intracellular calcium from either of the gametes in fertilization. Parallel experiments carried out with control spermatozoa capacitated in medium with low extracellular pH or high lactate substantiated the necessity of optimal sperm intracellular lactate levels, intracellular pH and calcium during sperm capacitation, for proper fertilization.

**Conclusions:**

This study confirms the importance of pyruvate/lactate metabolism in capacitating spermatozoa for successful fertilization, besides revealing for the first time the importance of sperm PDHc/ DLD in fertilization, via the modulation of sperm intracellular lactate, pH and calcium during capacitation. In addition, the observations made in the IVF studies in hamsters suggest that capacitation failures could be a plausible cause of unsuccessful fertilization encountered during human assisted reproductive technologies, like IVF and ICSI. Our studies indicate a role of sperm capacitation in the post-penetration events during fertilization.

## Introduction

Fertilization is a complex biological process, for which many of the prerequisites are still poorly understood. Fertilization success or failure depends on several sperm and egg factors [1]. Sperm capacitation too is an obligatory phenomenon for successful fertilization in mammals [2], [3]. Idiopathic fertilization failure in nature as well as during assisted reproductive practices such as conventional *in vitro* fertilization (IVF) has been attributed to problems of sperm capacitation [4], [5]; warranting molecular studies on the contribution of sperm capacitation to fertilization success.

Capacitation has been defined as the collection of biophysical and biochemical transformations, involving sperm metabolism, intracellular pH, intracellular cAMP, intracellular calcium concentration, intracellular ion concentrations, plasma membrane fluidity, membrane reorganization and reactive oxygen species [6]–[8]. The role of sperm metabolism in capacitation and eventually in fertilization has been an area of interest for over two decades [9], [10]. Recently, our laboratory, too has implicated pyruvate/lactate metabolism and the post-pyruvate metabolic enzymes, Pyruvate dehydrogenase complex (PDHc) and its E3 subunit dihydrolipoamide dehydrogenase (DLD) in the process of capacitation and acrosome reaction via the regulation of sperm intracellular lactate, intracellular pH (pH_i_) and intracellular calcium [Ca^2+^]_i_ [11]–[13]. Inhibition of PDHc/DLD was achieved by the use of the DLD-specific inhibitor, 5-methoxyindole-2-carboxylic acid (MICA). Downregulation of the PDHc/DLD activity in these MICA-treated (MT) hamster spermatozoa inhibited capacitation and acrosome reaction, with no significant effects on hyperactivation and tyrosine phosphorylation [11]. The mechanism of inhibition of capacitation and acrosome reaction in the MT-spermatozoa was worked out in the laboratory [13]. It was demonstrated that MT-spermatozoa showed lactate accumulation (due to PDHc/DLD inhibition and thus, pyruvate non-consumption), which resulted in lowering of initially, the intracellular pH and eventually, the intracellular calcium in these cells, causing blocked capacitation and acrosome reaction.

Deviation in this regulation resulting in sperm capacitation failure; is likely to affect the fertilization-competence of these spermatozoa. To validate this premise and understand the mechanism involved, we performed *in vitro* fertilization studies with spermatozoa; in which PDHc/DLD was inhibited by the use of the specific DLD inhibitor, 5-methoxyindole-2-carboxylic acid (MICA).These MICA-treated (MT-), non-capacitated spermatozoa, as anticipated, failed to fertilize the oocytes, thus, supporting the importance of sperm capacitation for successful fertilization. The results also substantiated the role of pyruvate/lactate metabolism in fertilization, in addition to establishing the requirement of a functional sperm PDHc/DLD in hamster fertilization.

## Materials and Methods

### Spermatozoa collection, *in vitro* capacitation and assessment of sperm hyperactivation

Male golden hamsters (*Mesocricetus auratus*) aged 6 months were used for the *in vitro* capacitation studies that involved modified TALP-PVA medium (Tyrode’s medium with albumin, lactate, pyruvate and polyvinyl alcohol) as described earlier [13]. Briefly, the caudae epididymidum were dissected out from anesthetized animals, rinsed in the medium, pierced with a fine needle and the released contents containing the spermatozoa was collected in the modified Tyrode’s medium. After a few minutes of incubation at 37°C, 5% CO_2_, a uniform suspension of spermatozoa was obtained which was then taken for a sperm count in a Makler chamber and a HTM-CEROS (Hamilton Thorne, Beverly, MA) computer assisted sperm analyzer (CASA). For *in vitro* fertilization (IVF) experiments; spermatozoa were collected after 3 h of capacitation in TALP-PVA medium and then used for inseminating the oocytes. MICA, the specific inhibitor of DLD, was dissolved in the TALP-PVA media as described earlier [11] and all the experiments were done with a 5 mM final concentration. The acrosome reaction was always assessed for MT- spermatozoa, to ensure that the inhibitor was working [13]. The present study was approved by the Institutional Animal Ethics Committee of the Centre for Cellular and Molecular Biology, Hyderabad, India.

Hamster sperm hyperactivation and the related motility kinematic parameters namely curvilinear velocity (VCL), linearity (LIN), amplitude of lateral head displacement (ALH) were assessed using CASA, according to the criteria described earlier [11]. The set up values of the CASA were as follows: frames acquired, 50; frame rate (Hz), 60; minimum contrast, 25; minimum cell size (pixels), 3; low average path velocity cut off (μm/sec), 7.5; medium average path velocity cut off (μm/sec), 12.5; low straight line velocity cut off (μm/sec), 5; static head intensity limits, 0.2–1.47; static head-size limits, 0.12–7.37;static elongation limits, 1–98; magnification, 1.43 (4x); video frequency (Hz), 60; bright field, off; slide temperature, 37°C; field selection mode, manual [11]. Based on these kinematic parameters, the non-hyperactivated spermatozoa (exhibiting planar motility pattern) could be differentiated from the hyperactivated spermatozoa (exhibiting either circular or helical motility patterns) using the SORT facility of the CASA. Spermatozoa with data points ≥15, VCL>300 µm/sec, LIN<40%, ALH>12 µm were sorted as hyperactivated (those exhibiting either circular or helical motility pattern) and spermatozoa with data points ≥15, VCL<300 µm/sec, LIN>40% and ALH<12 µm were sorted as non-hyperactivated spermatozoa (exhibiting planar motility pattern). A total of ∼100 individual spermatozoa were sorted at each time point to establish whether the spermatozoa were hyperactivated or not.

### Superovulation and oocyte collection

Three-month-old cyclic female hamsters were used in this investigation. On day 1 of the estrous cycle (confirmed by postovulatory discharge), before 10 a.m., ovarian hyperstimulation was induced by subcutaneous injection of 10 IU equine chorionic gonadotrophin (eCG -Folligon^®^; Intervet, Boxmeer, The Netherlands) and ovulation was induced by 10 IU human chorionic gonadotrophin (hCG-Chorulon^®^; Intervet, Boxmeer, The Netherlands) injected between 48–56 h after eCG injection [14]. Animals were anesthetized at 17±1 h after hCG injection. Oviducts were collected in a 35 mm dish (Nunc, Roskilde, Denmark) containing 1 ml TALP-PVA medium. The cumulus–oocyte complexes (COCs) were collected by gently teasing the ampulla region of the oviducts, and the COC mass was digested using hyaluronidase (1 mg/ml) and the cumulus-free zona intact oocytes were washed three times in TALP-PVA medium and incubated at 37°C in 5% CO_2_, under mineral oil (embryo-tested, Sigma,, St. Louis, MO, USA), until being used for IVF.

### *In vitro* fertilization

**Figure 1:**
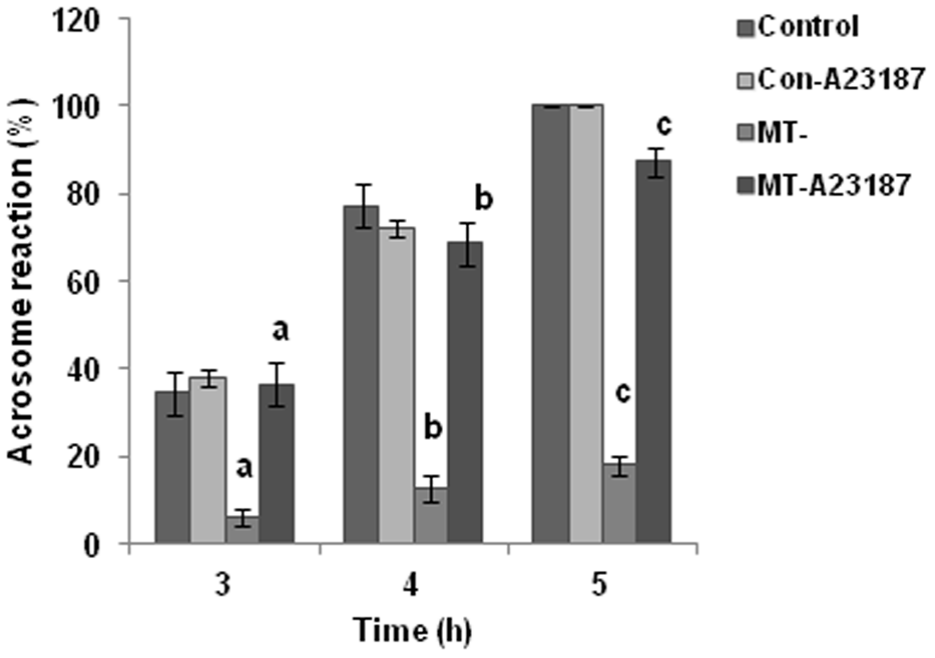
Acrosome reaction studies after induction with calcium ionophore, A23187. Induction of acrosome reaction was seen in MT- spermatozoa with 0.2 µM A23187, when evaluated at 3, 4 and 5 h of capacitation. Values with same superscript indicate statistically significant changes at p<0.05. Values represent mean ± SD.

Freshly collected oocytes (metaphase II-arrested, 10 oocytes per drop) were placed in a 100 µl fertilization drop of TALP-PVA medium under mineral oil and an aliquot of spermatozoa (final concentration of 10,000 – 20,000 spermatozoa, 2.5 µl) previously capacitated for 3 h (different capacitation conditions were used, as described under separate section) was added [14]. Co-incubation was carried out for at least 3 h at 37°C in 5% CO_2_ under mineral oil to prevent evaporation and pH changes. In all IVF experiments, spermatozoa were capacitated for 3 h under various conditions as indicated and then used for IVF, since in preliminary experiments it was established that in hamster spermatozoa, capacitation (as judged by the occurrence of acrosome reaction) begins at 3 h and reaches a peak by 5 h (Figure. 1).

After 3 h of co-incubation, the oocytes were washed in TALP-PVA medium to remove the excess bound spermatozoa, stained with Hoechst 33342 (30 µg/ml, Sigma, St. Louis, MO, USA) and their fertilization status was confirmed in the Axiovert microscope (Carl Zeiss Inc, Germany), 40x objective. The various cellular events monitored included meiotic plate reorganization, second polar body release and formation of both pronuclei. Only those oocytes that showed both 2^nd^ polar body release and pronuclei formation were scored as ‘properly fertilized’. Thirty to 50 oocytes from at least 4–7 different females were used for each determination. All experiments were repeated at least 4 times with spermatozoa from different males. All experiments were carried out with proper (solvent) control.

Irrespective of the media conditions for sperm capacitation, IVF was always done in the TALP-PVA medium. For alkalinization experiments, MT- spermatozoa were treated with 15 mM ammonium chloride (NH_4_Cl) from 0 h itself during capacitation and then used for IVF after 3 h. NH_4_Cl is routinely used for increasing the pH_i_ of spermatozoa [15]. For low pH studies, control spermatozoa were capacitated for 3 h in TALP-PVA medium, the pH of which was lowered to 6.8 and 7.0. For G media (TALP-PVA medium without pyruvate-lactate) studies, MT- spermatozoa were capacitated in G medium (MT-G) with and without 5 mM MICA and then used for IVF. In media which had only pyruvate-lactate (PL medium) and no glucose, spermatozoa were incubated for 3 h in this media before IVF. For experiments involving treatment with calcium ionophore, both control and MT- spermatozoa were treated for 5 minutes with 0.2 µM calcium ionophore, A23187 and then used for IVF. Calcium ionophore (Sigma, St. Louis, MO, USA) was prepared as a stock solution in DMSO and working dilutions were made in the TALP-PVA medium. 0.2 µM A23187, did not affect sperm motility drastically. It is known that a longer exposure of sperm to A23187 inhibits motility [16]. Acrosome reaction was assessed for control and MT-spermatozoa after the addition of A23187 at 3, 4 and 5 h of capacitation (Figure. 1).

Appropriate solvent and additives’ (NH_4_Cl, MICA, etc.) controls were always done alongside to ensure that these did not have an effect on fertilization outcome via their direct effect on oocytes. In these, co-incubation of oocytes and control spermatozoa was carried out in the presence of the additives (2.5 µl of 5 mM MICA / 5 mM MICA+15 mM NH_4_Cl / 5 mM MICA-G /5 mM MICA-G+5 mM NH_4_Cl / TL19 medium / pH 6.8 TALP medium /pH 7.0 TALP medium / 0.2 µM A23187 medium) and these conditions were further used for experiments with MT- spermatozoa, as the fertilization was found to be 100% (Table S1A). Control experiments were set up to also rule out the effect of the additives, NH_4_Cl and A23187 on the parthenogenetic activation of the hamster oocytes (Table S1B).

### Statistical analysis

ANOVA test with Tukey-Kramer multiple comparisons was performed to analyze the results statistically using the software Graph Pad, Prism, version 3.02. P values <0.05 were considered significant.

## Results

### Pyruvate/lactate alone in capacitating medium are sufficient to support fertilization

**Figure 2:**
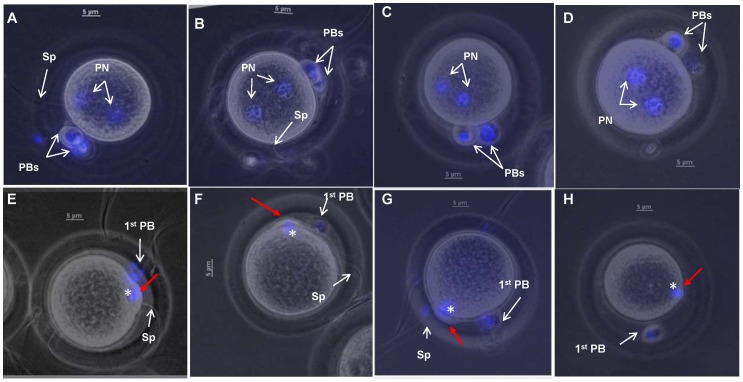
*In vitro* fertilization results with control (A-D) and MT- spermatozoa (E-H). Oocytes fertilized using control spermatozoa showed proper fertilization (PF) as judged by the presence of both polar bodies (PBs) and both pronuclei [PN].(A-D). Oocytes fertilized with MT-spermatozoa showed defective fertilization. In these spermatozoa, only meiotic spindle reorganization was visible (asterisk, E-H) and the 2^nd^ polar body extrusion had failed (red arrow in E-H). Oocytes were stained with Hoechst 33342 to visualize the polar bodies and pronuclei and the images presented are a merge of both brightfield and fluorescence. Magnification used was 400 x. Scale bars indicate 5 µm.

All the oocytes fertilized with hamster spermatozoa capacitated in TALP-PVA medium showed proper fertilization (Control), with both the polar bodies (PBs) and the pronuclei (PN) visible (Figure. 2A-D, Table 1). Hamster spermatozoa incubated in TALP medium devoid of glucose (PL medium) successfully fertilized all the oocytes (Control-PL), 100%, Table 1), indicating that the presence of pyruvate/lactate alone during capacitation is sufficient for hamster fertilization.

**Table 1:** Fertilization outcome with MICA-treated spermatozoa.

Sperm Treatment	Fertilization (%) #
Control	100±0 ^a^
Control-PL	100±0^b^
MT-	6.2±4.6 ^a, b^

Control: Control spermatozoa capacitated in TALP-PVA medium; Control-PL: spermatozoa capacitated in PL medium; MT-: MICA-treated spermatozoa in TALP-PVA medium.

# Values represent mean±SD.

Values with the same superscript differ significantly, p<0.05.

### Inhibiting pyruvate/lactate metabolism in spermatozoa affects fertilization: MICA-treated spermatozoa are unable to fertilize oocytes properly

Inhibiting PDHc/DLD affects pyruvate/lactate metabolism [13], since PDHc metabolizes pyruvate to acetyl CoA. Aberrant pyruvate/lactate metabolism during capacitation in these MT- spermatozoa resulted in only 6.2±4.6% oocytes showing proper fertilization (Table 1). The majority of these oocytes (∼90%) showed only meiotic plate reorganization (MPR, asterisk, Figure. 2E-H). These oocytes were also observed after 8 and 18 h and they maintained the same condition of MPR (data not shown). All oocytes inseminated with control spermatozoa (Control) showed 100% fertilization (Table 1).

### Reducing lactate load on the MICA-treated spermatozoa improves their fertilization ability

To ascertain if lactate accumulation in MT- spermatozoa [13] was responsible for the reduced fertilization, IVF was performed with MT- spermatozoa capacitated in G medium (MT-G). Reduced lactate load on these spermatozoa improved their fertilizing potential. The IVF results indicated that 35±6.7% oocytes were fertilized with MT-G spermatozoa (Table 2) in contrast to 6±4.2 % oocytes fertilized by MT spermatozoa.

**Table 2:** Fertilization with MICA-treated spermatozoa capacitated in G medium (MICA-treated-G) with or without ammonium chloride [NH_4_Cl].

Sperm Treatment	Fertilization (%) #
Control	100±0^a,b,c^
Control-G	100±0
MT-G	35±6.7^a,d,e,f^
MT-	6±4.2 ^b,d,g^
MT-15NH_4_Cl	75.5±3.3^b^
MT- G - 5NH_4_Cl	73±0.8^e,g,h^
MT- 5NH_4_Cl	1±2^c,f, h^

Control: Control spermatozoa in TALP-PVA medium; Control-G: control spermatozoa in G medium; MT-: MICA-treated spermatozoa; MT-G: MT spermatozoa in G medium; MT-15NH_4_Cl: MT spermatozoa in TALP-PVA medium alkalinized with 15 mM ammonium chloride; MT-G-5NH_4_Cl: MT-G spermatozoa alkalinized with 5 mM ammonium chloride; MT-5NH_4_Cl: MT- spermatozoa alkalinized with 5 mM ammonium chloride.

# Values represent mean±SD.

Values with the same superscript differ significantly at p<0.05.

### Alkalinization of the MICA-treated spermatozoa improves their fertilization potential

Increased lactate load decreases the pH_i_ of the MT spermatozoa [13] and thus, alkalinizing these spermatozoa was expected to improve the fertilization outcomes. Alkalinization of the male gamete resulted in 75.5±3.3 % of the oocytes showing proper fertilization, in contrast to 6±4.2% fertilization seen with untreated MT spermatozoa (Table 2). IVF done with MT-G spermatozoa alkalinized with only 5 mM NH_4_Cl (MT-G - 5NH_4_Cl) revealed fertilization success (73±2%) similar to MT-15NH_4_Cl (75.5±3.3 %) [Table 2].

### Control spermatozoa capacitated in TALP-PVA media with low pH or high lactate have compromised fertilizing ability

In order to assess if the accumulation of lactate and subsequent lower pH_i_ in the spermatozoa during capacitation [13] in general had detrimental effects on the outcome of hamster IVF (similar to that seen with MT spermatozoa), we carried out IVF with control spermatozoa capacitated in TALP-PVA medium supplemented with 19 mM lactate (normally TALP has 12.8 mM lactate) or with lower pH of 6.8 and 7.0. IVF with MT-spermatozoa was also done in parallel as a control for defective fertilization. IVF results indicated that all the above sperm treatments resulted in low success rate (49±5.1% oocytes for TL19; 38±6.9% for pH 6.8 and 44±8% for pH 7.0 spermatozoa) [Table 3].

**Table 3:** Fertilization with spermatozoa capacitated in TALP-PVA medium with high lactate and low pH (6.8 and 7.0).

Sperm Treatment	Fertilization (%)#
Control	100±0^a, b, c^
TL19	49±5.1^a^
pH 6.8	38±6.9^b^
pH 7.0	44±8.0^c^

Control: Control spermatozoa in TALP-PVA medium with pH 7.5 and 12.8 mM lactate; TL19: spermatozoa in TALP-PVA medium having 19 mM lactate; pH 6.8: spermatozoa in TALP-PVA medium having pH 6.8; pH 7.0: spermatozoa in TALP-PVA medium having pH 7.0.

# Values represent mean±SD.

Values with the same superscript differ significantly at p<0.05.

### Increasing [Ca^2+^]_i_ in MICA-treated spermatozoa during capacitation improves their fertilizing ability

**Figure 3:**
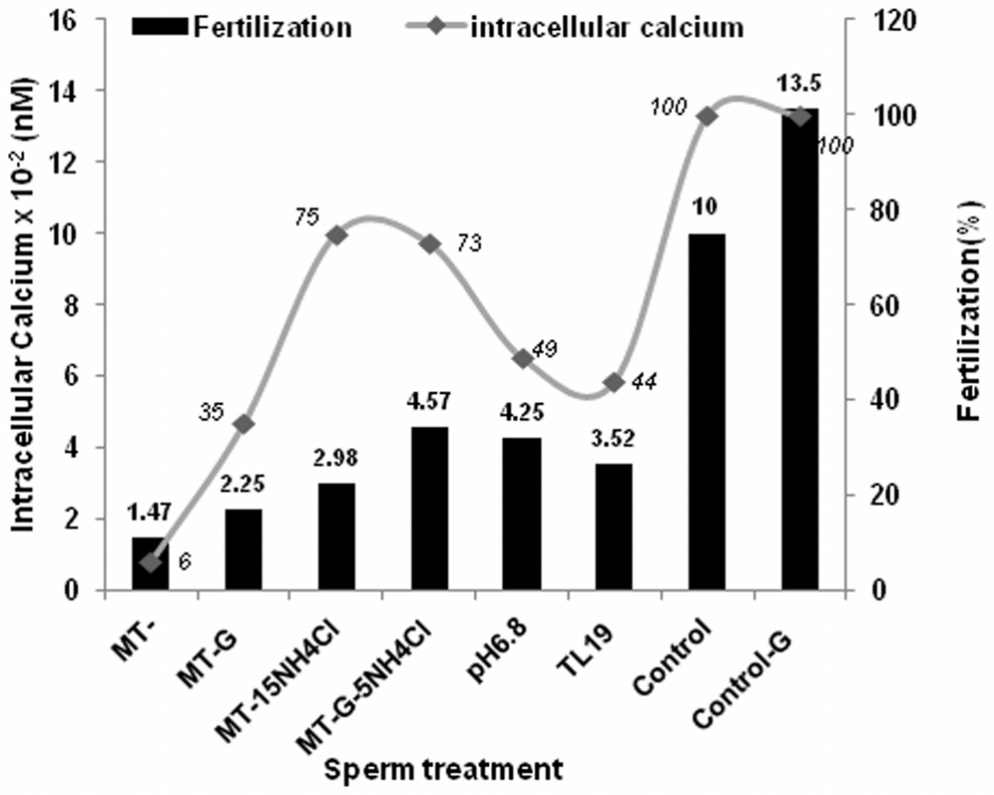
Graph showing the sperm intracellular calcium levels (nM) under different sperm treatments and the fertilization outcomes (%) [secondary axis].

Since [Ca^2+^]_i_ level in MT-spermatozoa was low [13], we envisaged that increasing calcium level in the MT spermatozoa would overcome the defective fertilization observed by these spermatozoa. Therefore, MT spermatozoa were treated at 2.55 h briefly (for 5 min) with 0.2 µM calcium ionophore, A23187 (MT-PreCa) before IVF. It was observed that oocytes fertilized with MT-PreCa spermatozoa showed 38±9.6% success as opposed to 6±4.6% in MT spermatozoa (Table 4). Acrosome reaction induction was also seen with this concentration of A23187 in MT-spermatozoa at all the 3 time points assessed (Figure. 1). A correlation (Spearman correlation coefficient, r = 0.8503, p<0.05) was seen between the sperm intracellular calcium levels [13] in the different treatments and the fertilization rates seen in this study (Figure. 3).

**Table 4:** Fertilization with MT-spermatozoa pretreated with calcium ionophore, A23187.

Sperm Treatment	Fertilization (%)
Control	100±0^a^
MT-	6±4.6^a,b,c^
MT-PreCa	38±9.6 ^b^
Post-fertilization	42.1±3.9^ c^

Control: Control spermatozoa in TALP-PVA medium; MT-: MICA treated spermatozoa; MT-PreCa: MT-spermatozoa pretreated with 0.2 µM calcium ionophore A23187; Post-fertilization: treatment of defective oocytes with 40 nM A23187, post-fertilization.

Values represent mean±SD.

Values with the same superscript differ significantly at p<0.05.

In another set of experiments with calcium ionophore, oocytes showing defective fertilization were treated with 40 nM A23187 for 10 minutes. It was seen that calcium ionophore treatment resulted in 42.1±3.7% of oocytes showing both, PB release and pronuclei formation, as compared to 6% fertilization seen in untreated MT-fertilized oocytes (Table 4).

## Discussion

This study confirms the importance of sperm capacitation for fertilization, in general and of sperm pyruvate/lactate metabolism in fertilization, in particular [10], [17]. Pyruvate/lactate are sufficient to support fertilization in hamsters, as demonstrated by IVF studies with spermatozoa capacitated in TALP medium devoid of glucose (Table 1). However, there is an optimum level of lactate/pyruvate required by the spermatozoon for it to remain fertile, since increase in the lactate load on the spermatozoa reduces its fertilizing potential (Table 3). Inhibiting pyruvate/lactate metabolism with PDHc/DLD inhibitor, MICA affected fertilization (Table 1). Since DLD mutant is embryonically lethal [18] making the use of DLD specific inhibitor, MICA is the best possible approach. The importance of pyruvate metabolism has been highlighted in embryo development, by the use of PDHA1 knockout model as well, where it was seen that oocytes compromised in PDHc activity (PDHA1 is another subunit of PDHc) fail to develop beyond the 1-cell zygote stage *in vivo* [19]. The authors hypothesized that this effect may be because of a “lactic acidosis-like condition”, which is created in the oocyte thereby affecting normal development. This brings into view the fact that pyruvate metabolism (via PDHc activity, since both DLD and PDHA1 are subunits of this complex) is important for fertilization and embryo development via pH_i_ regulation irrespective of the cell type (spermatozoon or oocyte) or the approach used (i.e. either DLD inhibition in our study or PDHA1 knockout in the study by Johnson *et al* [19]).

**Figure 4:**
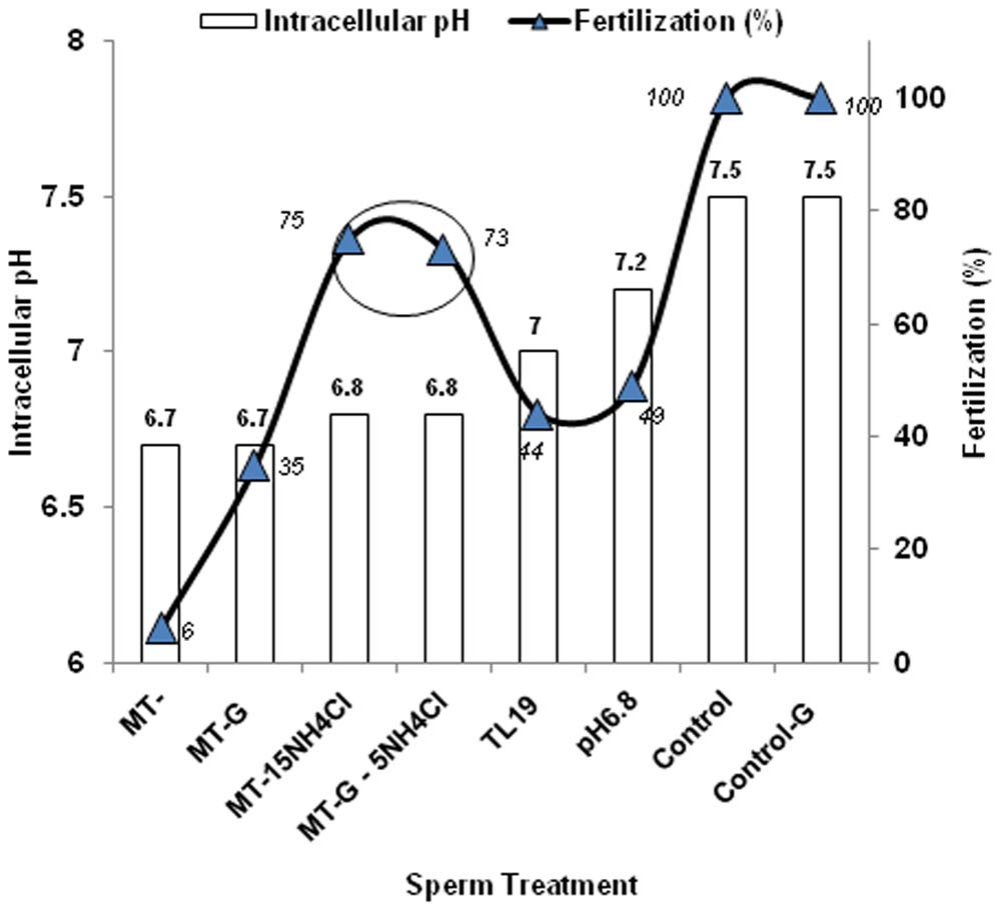
Graph showing the intracellular pH of spermatozoa (primary axis) under various sperm treatments and their corresponding fertilization outcomes (%) [secondary axis].

Inhibition of sperm DLD results in defective fertilization due to lactate accumulation in the spermatozoa, which in turns adversely affects sperm pH_i_ and [Ca^2+^]_i_ crucial for sperm capacitation [8], [20]–[25]. This high lactate, and low sperm pH_i_and [Ca^2+^]_i_ affects capacitation and acrosome reaction [13], and eventually the fertilizing potential of the spermatozoa, as seen in this study [Table 2, 3]. Improvement in the fertilization rate after increasing intracellular pH or calcium with NH_4_Cl [Table 2] and calcium ionophore, A23187, respectively [Table 4] supports the hypothesis that increasing intracellular pH/calcium improves capacitation/acrosome reaction in MT-spermatozoa, thereby eventually improving their fertilizing potential. This study on PDHc/DLD contributes to the knowledge available on the importance of sperm pH_i_ and [Ca^2+^]_i_ in mammalian fertilization [26]–[32]. A correlation (spearman correlation coefficient, r = 0.7683, p<0.05) was seen between the sperm pH_i_ [13] and the fertilization rate under the various experimental conditions studied, where low sperm pH_i_ resulted in lower fertilization rates (Figure. 4). Alkalinized MT and MT-G spermatozoa, showed a deviation in this trend (Figure. 4, encircled), suggesting a likely post-fertilization effect of the added NH_4_Cl to the fertilized oocyte. In fact, it was also seen that treatment of defectively fertilized oocytes with 2.5 mM NH_4_Cl post-fertilization resulted in 71±3% fertilization (Table S2) when compared to 6.2±4.6 % fertilization with untreated MT- spermatozoa.

**Figure 5:**
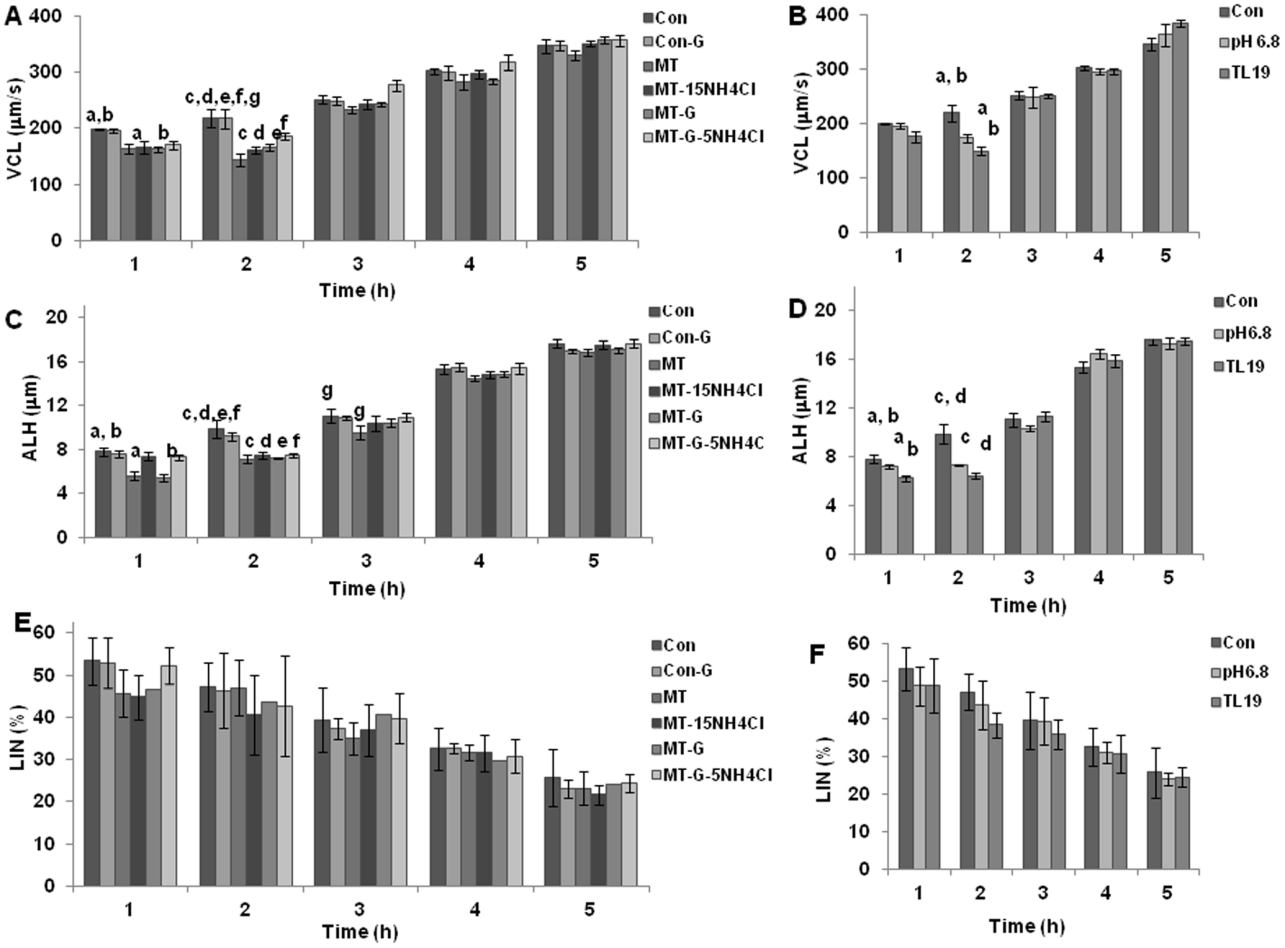
Assessment of sperm hyperactivation of spermatozoa under the various experimental conditions used. VCL (A and B), ALH (C and D) and LIN (E and F) were considered for assessing hyperactivation in the spermatozoa. Values with same superscript indicate statistically significant changes at p<0.05.

One another sperm characteristic, sperm hyperactivation (HA) is considered very important for mammalian fertilization [33], [34]. To assess if the various conditions used in this study affected sperm HA, which ultimately would have a bearing on the fertilization outcome, we assessed sperm HA during capacitation for the various conditions used (Figure. 5). It was seen that the effect was evident until 2 h of incubation in VCL (Figure. 5A and B), ALH (Figure. 5C and D) and LIN (Figure. 5E and F) parameters, after which the spermatozoa recovered. This trend with MICA has been shown earlier from our laboratory by Mitra and Shivaji [11]. Since the spermatozoa were picked up at 3 h of capacitation for IVF studies, it is possible that effects on HA may not influence IVF outcomes.

The oocyte resumes meiosis and becomes competent to begin embryonic development upon activation. The mammalian oocyte is activated in a fertilization-dependent manner. Oocyte activation is characterized by many events including changes in membrane to prevent polyspermy, release of the second meiotic arrest and completion of meiosis, posttranscriptional modifications of maternal mRNAs, and cytoskeletal rearrangements [35]. In this context, a careful observation of the oocytes fertilized with MT-spermatozoa revealed that the defective oocytes were arrested at the meiotic resumption step during oocyte activation. Subsequent to sperm penetration, initial occurrence of meiotic plate reorganization was observed; but the subsequent release of 2^nd^ polar body and pronuclei formation were not seen. Post-fertilization treatment of defectively-fertilized oocyte with calcium ionophore (Table 4) and NH_4_Cl (Table S2) resulted in 2^nd^ polar body release and pronuclei formation (both male and female), confirming sperm-penetration and ruling out the possibility of parthenogenetic activation. Oocytes activated parthenogenetically using ethanol showed 2 polar bodies and a single pronucleus (data not shown). Asch *et al* [36] report such observations of fertilization arrest in human IVF and in this context our study supports the hypothesis that such arrests could arise owing to defects in the spermatozoa and sperm capacitation, in particular. Although our study is in rodents [fertilization was inhibited in mouse species as well (unpublished results)], these results would help in understanding the clinical dilemma faced in unsuccessful human assisted reproductive technologies (ARTs).

Although sperm capacitation is an indispensable part of sperm fertility and is being studied for more than 60 years now, it is still accepted that “this process is not clearly understood”. The definition of capacitation has evolved over time (summarized by Ruffenach, 2009) [37] and in 1984, Chang [38] suggested that all processes leading up to the acrosome reaction should be referred to as the first part of sperm capacitation or in his original words “definition of capacitation should include all the events that lead to the development of the capacity of mammalian spermatozoa to *‘penetrate’* eggs.” It now seems essential to re-discuss the process of capacitation in the light of the interesting findings from this study.

We observe sperm penetration but not fertilization, (i.e. the subsequent events of activation, pronucleus formation, 2^nd^ polar body extrusion, etc.) in the case of MICA- treated/low pH spermatozoa. This reveals that these spermatozoa with improper capacitation have compromised fertility, in the post-penetration window. This is interesting because this highlights the importance of capacitation beyond penetration; thus, strengthening the hypothesis that fertilization failures can be due to paternal effects as highlighted in literature [39]–[42]. These studies point out that the failure to complete the fertilization process, syngamy or early cleavage could be the result of an early paternal effect. To validate and resolve this further in the context of humans, research on understanding the functional role of the male gamete beyond penetration, and unraveling the underlying causes of sperm pathology need to be carried out extensively [43], [44].

Adverse paternal effects on fertilization and embryo development could be due to centrosomal dysfunction, deficiency of oocyte-activating factors, failure of sperm head decondensation/damaged chromatin packaging, etc. [45]–[47]. These altered steps arising due to capacitation anomalies, cannot be ruled out. In the case of PDHc inhibition, sperm have reduced pH_i_ and elevated ROS [13], [48], which are likely to have a role in these altered sperm events, especially sperm chromatin packaging [49] and eventually fertilization. Besides these capacitation-associated anomalies, direct effects of “sperm acidification’ on oocyte activation/zygote development cannot be ruled out, although this hypothesis would require additional study.

Defectively fertilized oocytes showed completion of oocyte activation after treatment with calcium ionophore A23187, revealing that low [Ca^2+^]_i_ is responsible for the arrest seen and the MT- spermatozoa presumably fail to induce the calcium influx required for successful oocyte activation. Calcium signaling is crucial for fertilization [50] and it has been shown recently that calcium influx across the plasma membrane is mandatory for completion of meiosis; especially the extrusion of polar body in the metaphase II arrested oocytes [51] which are in accordance with our observations. It is evident that the molecular changes occurring during sperm capacitation pertinent for calcium influx and eventual oocyte-activation is compromised in the MT-spermatozoa, which are not capacitated properly. The mechanism, however, by which this happens, is not clear yet, but could be manifold as suggested by Barrosso et al [52], such as an improper localization of the oocyte activating factor PLC zeta due to improper capacitation [53]; untimely entry of spermatozoa into the oocyte due to delayed hyperactivation and penetration [54], compromised centriolar function, etc. Experiments to investigate these possibilities in the human and hamster spermatozoa are essential to understand how failed sperm capacitation due to low pH_i_ and [Ca^2+^]_i_ causes low calcium levels in fertilized oocytes and oocyte activation/fertilization failure.

In conclusion, this study has been an attempt to understand metabolic activities that regulate pH_i_ and calcium in sperm and modulate the capacitation-associated changes required for fertility. It highlights the role of the capacitation-associated, sperm metabolic proteins, PDHc/DLD in fertilization. Inhibition of sperm PDHc/DLD results in a “lactic acidosis- like condition’’ in the spermatozoa, where lactate, a common energy source turns unfavorable, upon exceeding its optimal limits and also affects sperm intracellular pH and calcium; thereby also highlighting the importance of pyruvate metabolism and lactate-pyruvate equilibrium during capacitation in the maintenance of sperm pH_i_, calcium and fertility. To the best of our knowledge, this appears to be for the first time that essentiality of sperm capacitation in the phenomenon of fertilization/ oocyte activation via pyruvate/lactate metabolism has been suggested. This observation would help in understanding the fertilization failure in human ARTs.

## Supporting Information

Table S1 A: Control IVF experiments set up with various additives. B: Control experiments done to study parthenogentic activation of oocytes.
(DOCX)

Table S2 Fertilization outcome on alkalinization of MT-fertilized oocytes with NH4Cl, post-fertilization.
(DOCX)
