# Supplementary material for: Inhibiting Sperm Pyruvate Dehydrogenase Complex and Its E3 Subunit, Dihydrolipoamide Dehydrogenase Affects Fertilization in Syrian Hamsters
Source: PLoS One. 2014 May 22;9(5):e97916. doi: 10.1371/journal.pone.0097916 (PMC4031208; doi:10.1371/journal.pone.0097916)
Supplement: Table S1 — A: Control IVF experiments set up with various additives. B: Control experiments done to study parthenogentic activation of oocytes. (DOCX) [file pone.0097916.s001.docx]

**Table S1A: Control IVF experiments set up with various additives**

| **Spermatozoa** | **IVF medium** | **Additives during**  **co-incubation** | **Fertilization (%)** |
| --- | --- | --- | --- |
| Control | TALP-PVA | 2.5 ul of 5mM MICA/5mM MICA in G medium | 100 ± 0 |
| Control | TALP-PVA | 2.5 ul of 5mM MICA + 15mM NH4Cl/ 5mM MICA in G medium + 5mM NH4Cl | 100 ± 0 |
| Control | TALP-PVA | 2.5 ul of TL19 medium | 100 ± 0 |
| Control | TALP-PVA | 2.5 ul of pH 6.8/7.0 medium | 100 ± 0 |
| Control | TALP-PVA | 0.2 µM 23187 | 100 ± 0 |

**Table S1B: Control experiments done to study parthenogentic activation of oocytes**

| **Medium for parthenogenesis studies** | **Additives in the medium** | **Activation seen** |
| --- | --- | --- |
| TALP-PVA | 2.5 ul of 15mM NH4Cl | No |
| TALP-PVA | 2.5ul of 0.2 uM A23187 | No |
