# Supplementary material for: Inhibiting Sperm Pyruvate Dehydrogenase Complex and Its E3 Subunit, Dihydrolipoamide Dehydrogenase Affects Fertilization in Syrian Hamsters
Source: PLoS One. 2014 May 22;9(5):e97916. doi: 10.1371/journal.pone.0097916 (PMC4031208; doi:10.1371/journal.pone.0097916)
Supplement: Table S2 — Fertilization outcome on alkalinization of MT-fertilized oocytes with NH4Cl, post-fertilization. (DOCX) [file pone.0097916.s002.docx]

**Table S2: Fertilization outcome on alkalinization of MT-fertilized oocytes with NH4Cl, post-fertilization**

| **Sperm Treatment** | **Fertilization (%) ^#^** |
| --- | --- |
| Control | 100 ± 0 ^a^ |
| MT- | 6.2 ± 4.6 ^a, b^ |
| Post fertilization- NH4Cl | 71 ± 3 ^b^ |

Control - control spermatozoa in TALP-PVA medium; MT- MICA - treated spermatozoa; Post-fertilization- NH4Cl: treatment of defective oocytes with 2.5 mM NH4Cl, post-fertilization

# Values represent mean ± SD.

Values with the same superscript differ significantly at p<0.05
